# IgE-Mediated Enhancement of CD4^**+**^ T Cell Responses in Mice Requires Antigen Presentation by CD11c^**+**^ Cells and Not by B Cells

**DOI:** 10.1371/journal.pone.0021760

**Published:** 2011-07-06

**Authors:** Frida Henningsson, Zhoujie Ding, Joakim S. Dahlin, Marius Linkevicius, Fredrik Carlsson, Kjell-Olov Grönvik, Jenny Hallgren, Birgitta Heyman

**Affiliations:** 1 Department of Medical Biochemistry and Microbiology, BMC, Uppsala University, Uppsala, Sweden; 2 Department of Immunology, Pathology and Genetics, Uppsala University, Uppsala, Sweden; 3 National Veterinary Institute, Uppsala, Sweden; Albany Medical College, United States of America

## Abstract

IgE antibodies, administered to mice together with their specific antigen, enhance antibody and CD4^+^ T cell responses to this antigen. The effect is dependent on the low affinity receptor for IgE, CD23, and the receptor must be expressed on B cells. *In vitro*, IgE-antigen complexes are endocytosed via CD23 on B cells, which subsequently present the antigen to CD4^+^ T cells. This mechanism has been suggested to explain also IgE-mediated enhancement of immune responses *in vivo*. We recently found that CD23^+^ B cells capture IgE-antigen complexes in peripheral blood and rapidly transport them to B cell follicles in the spleen. This provides an alternative explanation for the requirement for CD23^+^ B cells. The aim of the present study was to determine whether B-cell mediated antigen presentation of IgE-antigen complexes explains the enhancing effect of IgE on immune responses *in vivo*. The ability of spleen cells, taken from mice 1–4 h after immunization with IgE-antigen, to present antigen to specific CD4^+^ T cells was analyzed. Antigen presentation was intact when spleens were depleted of CD19^+^ cells (i.e., primarily B cells) but was severely impaired after depletion of CD11c^+^ cells (i.e., primarily dendritic cells). In agreement with this, the ability of IgE to enhance proliferation of CD4^+^ T cells was abolished in CD11c-DTR mice conditionally depleted of CD11c^+^ cells. Finally, the lack of IgE-mediated enhancemen of CD4^+^ T cell responses in CD23^-/-^ mice could be rescued by transfer of MHC-II-compatible as well as by MHC-II-incompatible CD23^+^ B cells. These findings argue against the idea that IgE-mediated enhancement of specific CD4^+^ T cell responses *in vivo* is caused by increased antigen presentation by B cells. A model where CD23^+^ B cells act as antigen transporting cells, delivering antigen to CD11c^+^ cells for presentation to T cells is consistent with available experimental data.

## Introduction

Apart from initiating various defense mechanisms via Fc-receptor binding or complement activation, antibodies have the capacity to regulate the immune responses against the antigen they are specific for [1]. This phenomenon is called antibody feedback regulation and has been known for over a century [2], although the molecular mechanisms behind are still not completely understood [1]. The most well known type of antibody feedback regulation is the ability of IgG to suppress antibody responses against large particulate antigens such as erythrocytes. This has been used successfully in the clinic since the 1960's to prevent Rhesus negative women from becoming immunized against fetal Rhesus positive erythrocytes, transferred via transplacental hemorrhage [3], [4]. In other situations, antibodies can enhance antibody responses. IgG has a dual role and, depending on subclass, enhances responses to protein antigens via Fc-gamma-receptors [5] or complement [6]. IgM is another well known enhancer of antibody responses, which utilizes the complement system [7], [8]. An interesting feedback circuit is the one initiated by IgE antibodies. TNP (trinitrophenyl)-specific IgE administered intravenously (i.v.) to mice together with small protein antigens such as BSA (bovine serum albumin)-TNP or OVA (ovalbumin)-TNP induce a several 100-fold higher primary antibody response than does antigen administered alone [9], [10], [11], [12]. The effect is most pronounced on the IgG response and formation of germinal centers and recall responses are also enhanced [13], [14], [15]. The enhancing effect of IgE on antibody responses is completely dependent on the presence of CD23, the low affinity receptor for IgE [9], [10], [16]. Murine CD23 exists in two isoforms, CD23a and CD23b. CD23a is constitutively expressed on B cells and follicullar dendritic cells (FDC), but not on dendritic cells [17], [18], and is the isoform involved in IgE-mediated enhancement of antibody responses [12]. The CD23b isoform has been found on enterocytes in the intestine and recently also on lung epithelial cells [19], [20]. It is well established by several independent groups that human as well as mouse B cells take up IgE-antigen complexes via CD23 and present the antigenic peptides to CD4^+^ T cells *in vitro* [21], [22], [23], [24], [25]. In analogy with these findings, TNP-specific IgE administered with OVA-TNP to mice enhances proliferation and activation of OVA-specific CD4^+^ T cells *in vivo* [15], [16]. The effect on T cells as well as on antibody responses requires that CD23 is expressed on B cells [11], [16]. These findings are compatible with the idea that the enhancing effect of IgE on antibody and T cell responses *in vivo* is explained by B cell-mediated antigen presentation. Whether B cells are able to present antigen to naïve T cells or not has been debated, and there is experimental support both in favour of [26], [27], [28], [29], [30] and against [31], [32], [33], [34] this idea. In a previous study, using the same experimental approach as in the present one, we followed the transport of IgE-antigen complexes in vivo [15]. IgE-antigen was found on the majority of B cells in the blood ten minutes after immunization and were detected in the splenic follicles on CD23^hi^CD21^dim^ cells (follicular B cells) after 30 minutes [15]. This observation provided an alternative explanation for the requirement of CD23^+^ B cells, suggesting that the enhancing effect of IgE on immune responses could be caused by concentrating the antigen to B cell follicles. The findings prompted us to ask whether CD23^+^ B cells are required both for transport and presentation of IgE-antigen complexes or whether they primarily act to transport the antigen. The results argue against the idea that presentation of IgE-antigen by CD23^+^ B cells is the explanation for IgE-mediated enhancement of CD4^+^ T cell responses *in vivo*.

## Materials and Methods

### Ethics statement

All animal experiments were approved by Uppsala Animal Research Ethics Committee (protocol numbers C117/7 and C146/10). The mice were bred and maintained in the animal facilities at the National Veterinary Institute (Uppsala, Sweden). Skilled personnel under the supervision of the veterinarian in charge routinely observed the health status of the mice.

### Mice

C57BL/6 and CD11c-DTR mice were obtained from Jackson Laboratories (Bar Harbor, ME), BALB/c mice from Bommice (Ry, Denmark), CD23-deficient (CD23^-/-^) mice were a kind gift from Dr Kishimoto [10] and were backcrossed to BALB/c for 10 generations [16]. DO11.10 mice [35] were obtained from Dr Westerberg, Karolinska Institute, Stockholm. DO11.10 mice are BALB/c mice that carry transgenic TCR α and β chains, resulting in a TCR recognizing OVA_323-339_ together with MHC class II I-A^d^. CD11c-DTR mice are BALB/c mice transgenic for a high affinity human diphtheria toxin receptor expressed under the *cd11c* promoter [36].

Offspring from heterozygous CD11c-DTR mated with wildtype BALB/c mice were used and DNA was extracted from the tail tip by digestion in 40 µl 1x modified Gitschier buffer (67 mM Tris-HCl (pH 8.8), 0.166 mM (NH_4_)_2_SO_4_, 6.5 mM MgCl_2_) with 1% 2-mercaptoethanol and 0.5% Triton X-100 at 95°C for 5 min. Thereafter, 0.5 mg/ml proteinase K (Qiagen, Hilden, Germany) was added and the digesting tissue was incubated at 55°C for 1 h followed by a 5 min incubation at 95°C. Residual tissue were removed by centrifugation at 16 000 x g for 2 min. This DNA was used in a PCR reaction with the following primers: DTR1, 5′-GCCACCATGAAGCTGCTGCCG-3′; DTR2, 5′-TCAGTGGGAATTAGTCATGCC-3′. Gene amplification was performed using reagents from Applied Biosystems (Carlsbad, CA) in a 25 µl PCR reaction mixture containing 2.5 µl 10x PCR buffer, 4 mM MgCl_2_, 0.2 mM dNTPs, 0.4 µM each of primers and 0.06 U/ml AmpliTaq DNA polymerase (4 min, 94°C; 15 s, 95°C; 1 min 63.1°C; 15 s 72°C, repeat previous steps for 35 cycles; 5 min 72°C). PCR products was electrophoresed on a 1.5 % agarose gel in 1x Tris-acetate-ethylenediaminetetraacetic acid (TAE) buffer at 100 V. The transgenic diphtheria receptor (DTR) gives a 625 bp band. For depletion of CD11c^+^ cells *in vivo*, CD11c-DTR mice were injected i.p. with 100 ng diphtheria toxin (Sigma-Aldrich) in 200 µl PBS. Mice were matched for age and sex within each experiment and were at least 6 weeks old.

### Antigens

OVA (ovalbumin) and TNP (picrylsulfonic acid/hydrate) were obtained from Sigma-Aldrich (St Louis, MO). TNP was coupled to OVA in 0.28 M cacodylate buffer, pH 6.9 as described [37]. The coupling reaction was performed at room temperature for 90 min and stopped by addition of an excess of glycyl-glycine (1 mg/ml, Merck, Darmstadt, Germany). Free TNP was removed by dialyzing against PBS. The number of TNP residues/OVA was determined [37] and batches with a TNP to OVA ratio between 1 and 2 were used with similar results.

### Antibodies

Monoclonal murine IgE-anti-TNP was derived from the B cell hybridoma IGELb4 [38] and purified and stored as described [16]. For flow cytometry we used PE-labeled anti-CD4 mAbs (GK1.5), APC-labeled anti-CD11c (HL3), PE-labeled MHC-II I-A^d^ (AMS-32.1), FITC-labeled anti-CD19 (1D3) and PE-labeled anti-B220 (RA3-6B2), all from BD Biosciences (San Jose, CA). The DO11.10 transgenic T cells were detected with FITC-labeled KJ1-26 mAb (Caltag Laboratories, Burlingame, CA) specific for this particular TCR heterodimer [39]. For confocal microscopy, FITC-labled anti-B220 (RA3-6B2), biotinylated KJ1-26, and APC-labeled streptavidin were used (all from eBioscience, San Diego, CA).

### Immunizations

Mice were immunized with purified IGELb4 (IgE-anti-TNP) [38] and OVA-TNP in the tail veins in a total volume of 200 µl PBS. IgE and antigen were pre-mixed in a test tube at room temperature and injected within 1 h. Amounts of IgE and antigen are detailed in the figure legends.

### Flow cytometry

Single cell suspensions from spleens were treated with ACK lysing buffer (0.15 M NH_4_CL, 1.0 M KHCO_3_, 0.1 mM Na_2_EDTA, pH 7.3) for 3 min to remove erythrocytes, washed in PBS and resuspended in FACS buffer (PBS with 2% fetal bovine serum). 5×10^5^ cells were stained at 4°C for 30 min in 100 µl FACS buffer with predetermined optimal amounts of labeled antibodies. Cells with the forward- and side-scatter properties of lymphocytes (for analysis of DO11.10 T cells) or all live cells (for analysis of CD11c^+^ cells) were collected on a FACScan cytometer or a LSR II cytometer (both from BD Biosciences) and analyzed using the FLOWJo Software. 2−3×10^5^ events were analysed. OVA-specific DO11.10 T cells were identified as CD4^+^KJ126^+^ and dendritic cells as CD11c^+^MHC-II^hi^.

### Isolation of cells

**Figure 1:**
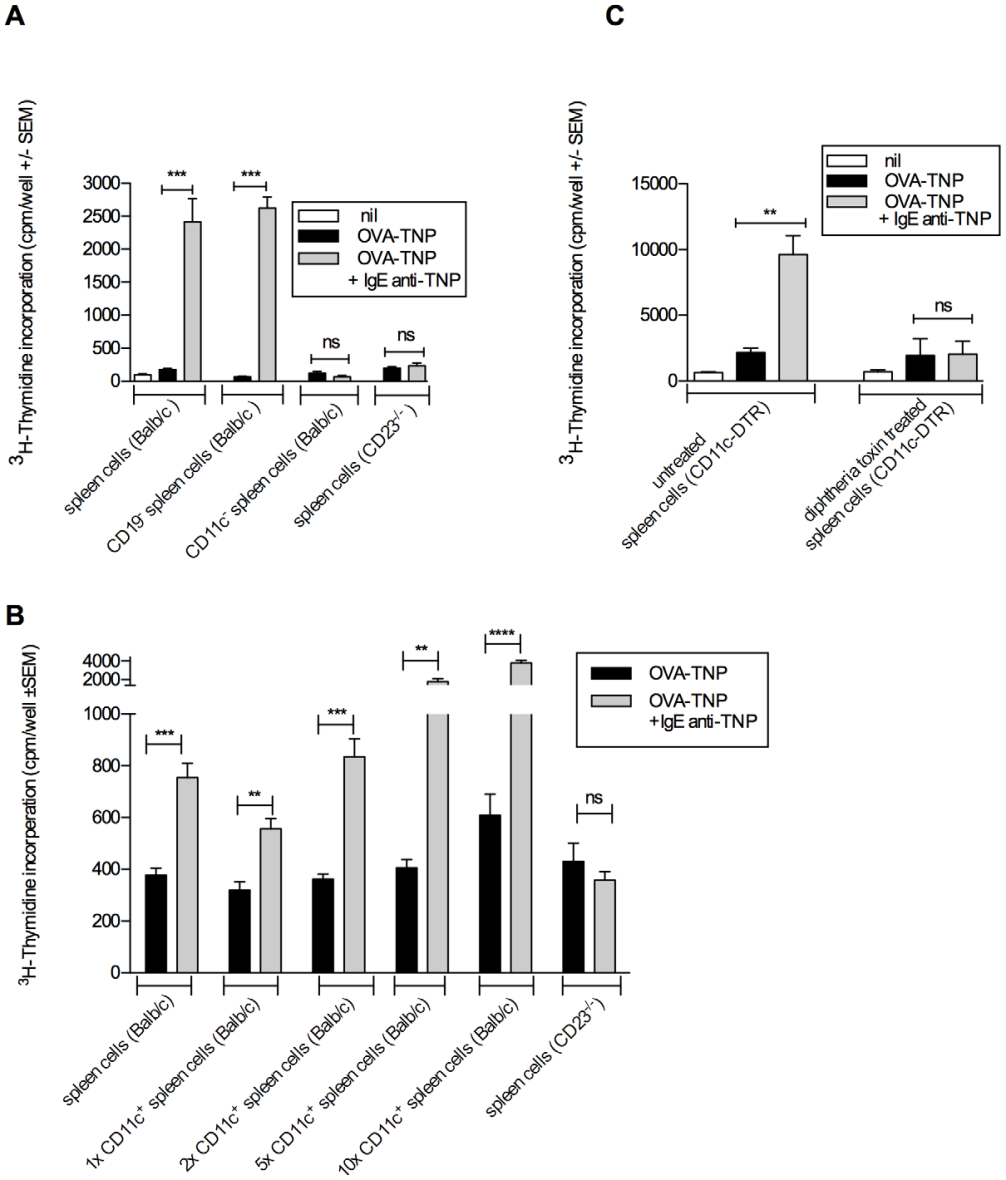
CD11c^+^ cells present IgE-antigen to CD4^+^ T cells *ex vivo*. A & B) BALB/c or CD23^-/-^ mice were immunized with 100 µg OVA-TNP alone or together with 250 µg of IgE anti-TNP. After 4 h, spleens were removed, fractionated, and tested for ability to activate CD4^+^ OVA-specific DO11.10 T cells *ex vivo.* C) CD11c-DTR mice were treated with 100 ng diphtheria toxin or left untreated. After 24 hours, the mice were immunized as in A and 4 hours later spleen cells were removed and tested for ability to activate DO11.10 T cells *ex vivo*. A) is representative of two identical and 4 similar experiments, B) of three similar experiments and C) of 5 identical and 2 similar experiments.

The MACS system (Miltenyi Biotech, Bergisch Gladbach, Germany) was used to obtain cell suspensions depleted of, or enriched for, defined populations. Single cell suspensions from spleens were treated with ACK as described above, resuspended in MACS buffer (PBS containing 0.5% bovine serum albumin and 2 mM EDTA) and labeled with anti-CD4-conjugated magnetic beads and CD4^+^ cells were collected using LS columns according to the manufacturer's instructions. The isolated cell population was >96% CD4^+^ as determined by flow cytometry. Spleen cells were depleted of CD11c^+^ cells by mixing with anti-CD11c-conjugated magnetic beads followed by passage over an LD column (which is specialized for depletion of cells): more than 94% of CD11c^+^MHC-II^hi^ cells were depleted (CD11c^−^ population in Figure 1A). To enrich for CD11c^+^ cells (Figure 1B) spleen cells were mixed with anti-CD11c-conjugated magnetic beads and passed over an LS column (which is specialized for enrichment of cells). Cells eluted from the column were >68% CD11c^+^. To deplete for B cells (Figure 1A), spleen cells were mixed with anti-CD19-conjugated magnetic beads followed by passage over an LD column. The flow through cells were >98% CD19^−^. To enrich for B cells, the fact that all leukocytes except mature and immature resting B cells express CD43 was used: spleen cells were mixed with anti-CD43-conjugated magnetic beads, passed over an LD column and the CD43^−^ flow through cells (>98% CD19^+^) were used as B cells in transfer experiments.

### Adoptive transfers

2.3−5.0×10^6^ CD4^+^ cells from DO11.10 spleens (positively selected as described above) and when applicable 17×10^6^ CD43^−^ B cells from BALB/c or C57BL/6 (negatively selected as described above) were adoptively transferred i.v. in 200 µl PBS.

### ^3^H-thymidin incorporation assay

BALB/c or CD23^-/-^ mice were immunized i.v. with 100 µg of OVA-TNP in PBS with or without 250 µg of IgE anti-TNP. Controls were left unimmunized. After 4 hours, spleens from BALB/c mice were removed and spleen cells were divided into three fractions: one was left unfractionated, one was depleted of CD19^+^ cells and one was depleted of CD11c^+^ cells whereas CD23^-/-^ spleen cells were left unfractionated (Figure 1A). In Figure 1B, CD11c^+^ cells were enriched on LS columns (see above). Unfractionated spleen cells were used as APC at a concentration of 6×10^5^ cells/well. When fractionated APC populations were used (Figures 1A & B), they were adjusted to the same volume as before fractionation to contain the same numbers of the respective APC as did unfractionated spleen cells. In Figure 1B, we also used 2x, 5x and 10x the number of APC as in the unfractionated populations. APC were added in 100 µl f-DMEM (National Veterinary Institute, Uppsala, Sweden) supplemented with 100 U/ml penicillin, 100 µg/ml streptomycin, 2 mM L-glutamine, 50 µM β-mercaptoethanol, and 5% heat-inactivated fetal bovine serum (all from Sigma-Aldrich) to 96 well tissue culture plates (Sarstedt, Nümbrecht, Germany) and irradiated (10 Gy). 1×10^5^ CD4^+^ DO11.10 responder cells in 100 µl f-DMEM were added to each well. After 48 h the cells were pulsed with 1 µCi ^3^H-thymidine (American Radiolabeled Chemicals, St Louis, MO) for 18–24 h. Incorporation of ^3^H-thymidine was measured in a beta counter (Wallac 1450 MicroBeta Trilux, Perkin Elmer, Waltham, MA). Mean and SEM were calculated from five replicate wells.

### Confocal microscopy

Tissue sections were prepared as described previously [15]. Briefly, spleens were frozen and embedded in O.C.T. compound (Sakura Finetek, Alphen aan den Rijn, The Netherlands) and 7 µm sections were cut using a cryostat. Air-dried sections were collected onto glass microscope slides (Mentzel-Gläser, Braunschweig, Germany) and stored frozen at −70°C. The slides were fixed in ice cold 50% acetone for 30 s and then in 100% acetone for 5 min. Rehydration of the sections was performed in PBS for 15 min at room temperature. Sections were then blocked with 5% horse serum (Sigma-Aldrich) in PBS for 1 h. After pouring off blocking solution, 2 µg/ml FITC-labeled anti-B220 and 1 µg/ml biotinylated KJ1-26, diluted in 100 µl blocking solution, was added to each section and left for 1 h at room temperature. After washing twice in PBS for 5 min, 100 µl of 2 µg/ml APC-labeled streptavidin was added, sections were stained for 1 h and washed twice in PBS. Slides were mounted in Fluoromount G (Southern Biotech, Birmingham, AL) and immunofluorescence was detected by a LSM 700 confocal microscope from Carl Zeiss (Thornwood, NY). Photos of every T cell zone on four non-consecutive sections from each individual spleen were taken by Zen 2009 software (Carl Zeiss). Numbers of KJ1-26^+^ T cells in each T cell zone were automatically quantified using ImageJ software (NIH, Bethesda, MD).

### Statistical analysis

Statistical differences between groups were determined by Student's *t*-test. Values for *p*>0.05 (not significant, ns), *p*<0.05 (*), *p*<0.01 (**) or *p*<0.001 (***) are indicated in the figures.

## Results

### CD11c^+^ cells present IgE-antigen to CD4^+^ T cells *ex vivo*

**Figure 2:**
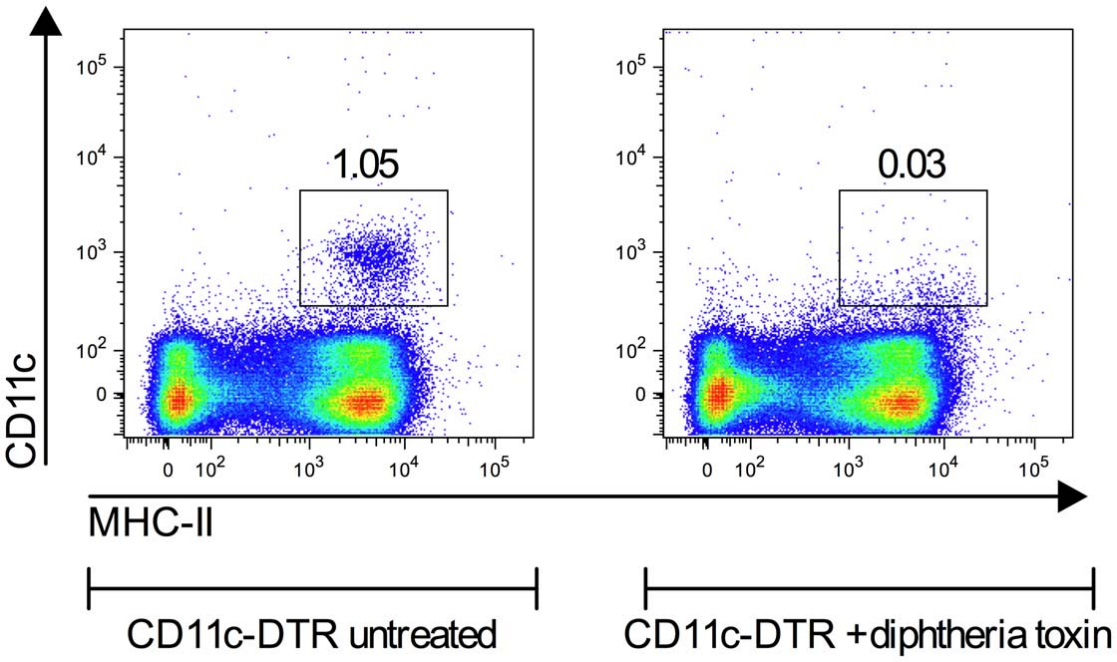
Diphtheria toxin depletes CD11c-DTR mice of CD11c^+^ cells. CD11c-DTR mice were treated with 100 ng diphtheria toxin or left untreated. Twenty-four hours later total spleen cells were analyzed in flow cytometry and dendritic cells were defined as CD11c^+^, MHC-II^hi^ cells. The numbers show % of live cells in a representative experiment with 97% depletion of dendritic cells.

To investigate whether B cells (CD19^+^) or CD11c^+^ cells present IgE-antigen complexes *in vivo*, mice were immunized with OVA-TNP alone or together with IgE-anti-TNP. Four hours later their spleens were removed, cell suspensions prepared, irradiated and used as APC (antigen presenting cells) *ex vivo*. Spleen cells removed after 1, 2, 3, and 4 h could induce efficient antigen presentation, with the best effect seen with APC removed after 4 h (data not shown). Spleen cells removed after 13 h had lost their capacity to present antigen *ex vivo* (data not shown). No additional antigen or immune complexes were added to the cell cultures, ascertaining that only OVA-peptides acquired *in vivo* were presented on MHC-II on the various APC populations. As responder cells, CD4^+^ T cells from DO11.10 mice were used. These T cells have a transgenic TCR recognizing OVA_323–339_ together with MHC class II I-A^d^ [35]. Unfractionated spleen cells from mice immunized with IgE-antigen complexes induced much more efficient T cell proliferation than spleen cells from mice given OVA-TNP alone whereas spleen cells from CD23^-/-^ mice could not induce T cell proliferation (Figure 1A). Interestingly, spleen cells depleted of B cells (CD19^−^) were equally efficient as unfractionated spleen cells, whereas spleen cells depleted of CD11c^+^ cells (CD11c^−^) completely lost their antigen presenting capacity, although B cells were present in this population (Figure 1A). In the reciprocal approach, enriching spleen cells for CD11c^+^ cells, we found that CD11c^+^ cells retained the antigen presenting capacity of unfractionated spleen cells (Figure 1B). Upon increasing the number of CD11^+^ cells up to 10-fold of the number present amongst the unfractionated spleen cells, also the T cell proliferation increased dramatically (Figure 1B). As an alternative approach, CD11c-DTR mice, expressing the high affinity receptor for diphtheria toxin under the CD11c promoter, were used as a source of APC in the *ex vivo* antigen presenting assay. CD11c-DTR mice can be conditionally depleted of cells expressing high amounts of CD11c by treatment with diphtheria toxin [36] (Figure 2). Toxin-treated and untreated CD11c-DTR mice were immunized with OVA-TNP alone or in complex with IgE anti-TNP. Four hours later their spleens were removed, spleen cells prepared, irradiated and used as APC in the T cell proliferation assay as described above. When total spleen cells from untreated CD11c-DTR mice immunized with IgE-antigen complexes were used as APC, they induced efficient T cell proliferation (Figure 1C). In contrast, APC derived from the spleens of toxin-treated CD11c-DTR mice were unable to induce T cell proliferation (Figure 1C). Taken together, these observations suggest that CD11c^+^ cells, but not B cells, in mice immunized with IgE-OVA complexes present antigen efficiently enough to induce proliferation of specific CD4^+^ T cells *ex vivo*.

### IgE-mediated enhancement of T cell proliferation *in vivo* depends on CD11c^+^ cells

**Figure 3:**
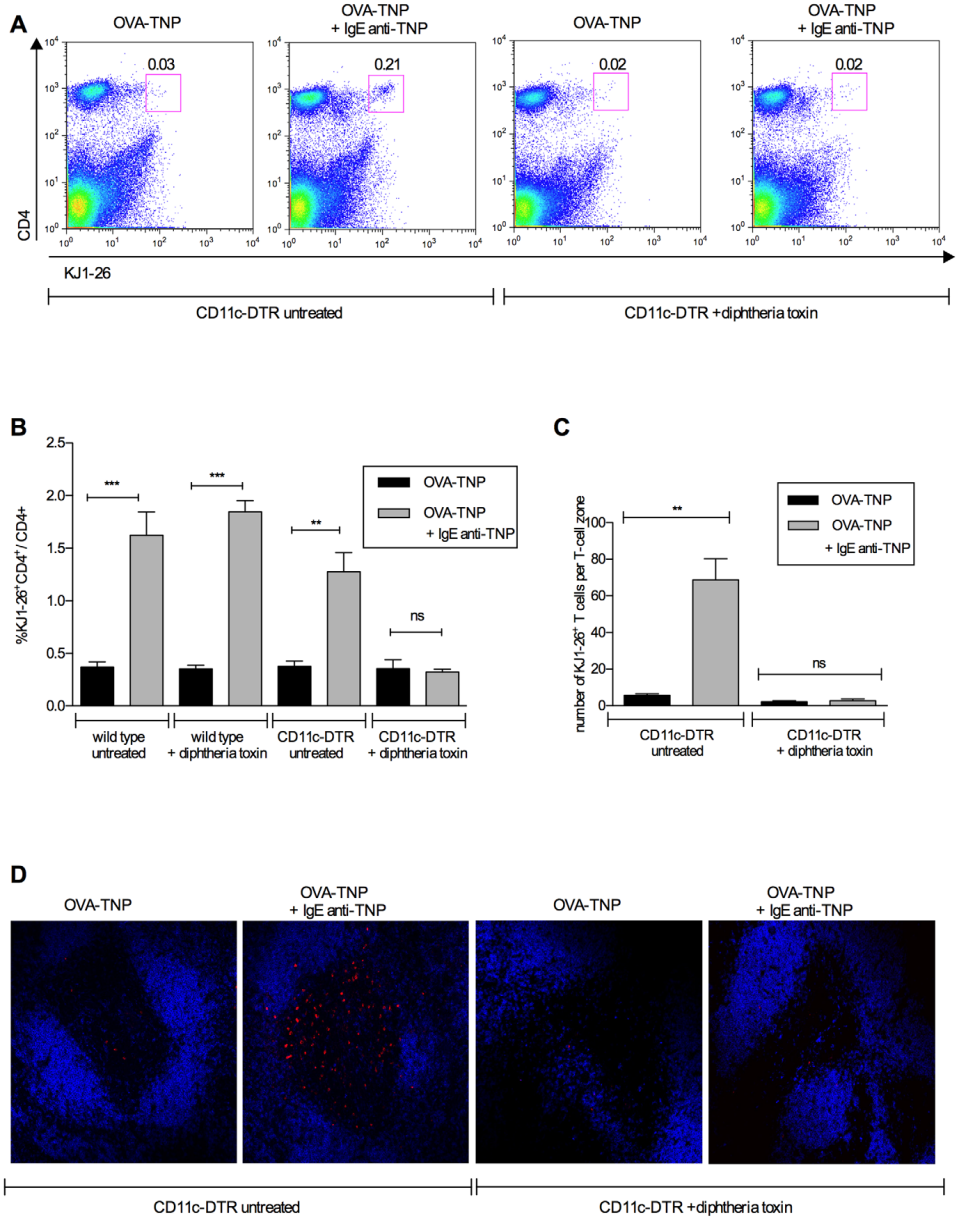
IgE-mediated enhancement of T cell proliferation *in vivo* is dependent on CD11c^+^ cells. CD11c-DTR mice, and in B) also wild type littermates, were treated with 100 ng diphtheria toxin or left untreated. The same day, the mice were transferred i.v. with 2.3−3.0×10^6^ CD4^+^ DO11.10 spleen cells. Twenty-four hours after diphtheria toxin treatment, mice were immunized i.v. with 20 µg OVA-TNP with or without 50 µg IgE anti-TNP. Three days later, spleens were analyzed for CD4^+^ KJ1-26^+^ T cells by flow cytometry (A, B) or for KJ1-26^+^ cells in confocal microscopy (C, D). A) Representative dot-plot of flow cytometry data; values represent % CD4^+^KJ1-26^+^ cells of gated lymphocytes. B) Mean values ± SEM of % CD4^+^KJ1-26^+^ of CD4^+^ cells analyzed in flow cytometry (3 mice/group) C) Mean values ± SEM of KJ1-26^+^ cells in non-consecutive sections of T cell zones analyzed by confocal microscopy (3 mice/group; 3–12 T cell zones/mouse). D) Visualization of representative T cell zones from each group: KJ1-26^+^ cells (red) and B220^+^ cells (blue). B) is representative of 4 experiments and C) of 1 experiment.

The conclusion from Figure 1 was deduced from an *ex vivo* antigen presentation assay. Next, we analyzed the ability of IgE-antigen complexes to induce T-cell proliferation directly *in vivo* in mice lacking CD11c^+^ cells. CD11c-DTR mice, and in some cases littermate controls, were treated with diphtheria toxin leading to depletion of 97% of CD11c^+^ spleen cells in the CD11c-DTR mice (Figure 2). Mice were then adoptively transferred with DO11.10 T cells and immunized. Three days later the number of splenic OVA-specific CD4^+^ T cells were analyzed, either in flow cytometry (Figure 3A, B) or in confocal microscopy (Figure 3C, D). IgE enhanced T cell proliferation in wildtype littermates equally well whether the mice had been treated with diphtheria toxin or not, showing that the toxin itself did not suppress T cell responses (Figure 3B, bars 1–4). In untreated CD11c-DTR mice, IgE also enhanced T cell proliferation (Figure 3B, bar 5 and 6). In sharp contrast, the ability of IgE to enhance T cell responses was completely abolished in CD11c-DTR mice that had been depleted of CD11c^+^ cells by toxin treatment (Figure 3B, bar 7 and 8). Analysis by an alternative approach, counting specific T cells in spleen sections, gave similar results (Figure 3 C,D). These data, strengthen the *ex vivo* data (Figure 1), and show that IgE-mediated enhancement of CD4^+^ T cell responses *in vivo* requires the presence of CD11c^+^ cells.

### MHC-II-incompatible B cells rescue IgE-mediated enhancement of T cell-proliferation in CD23^-/-^ mice

**Figure 4:**
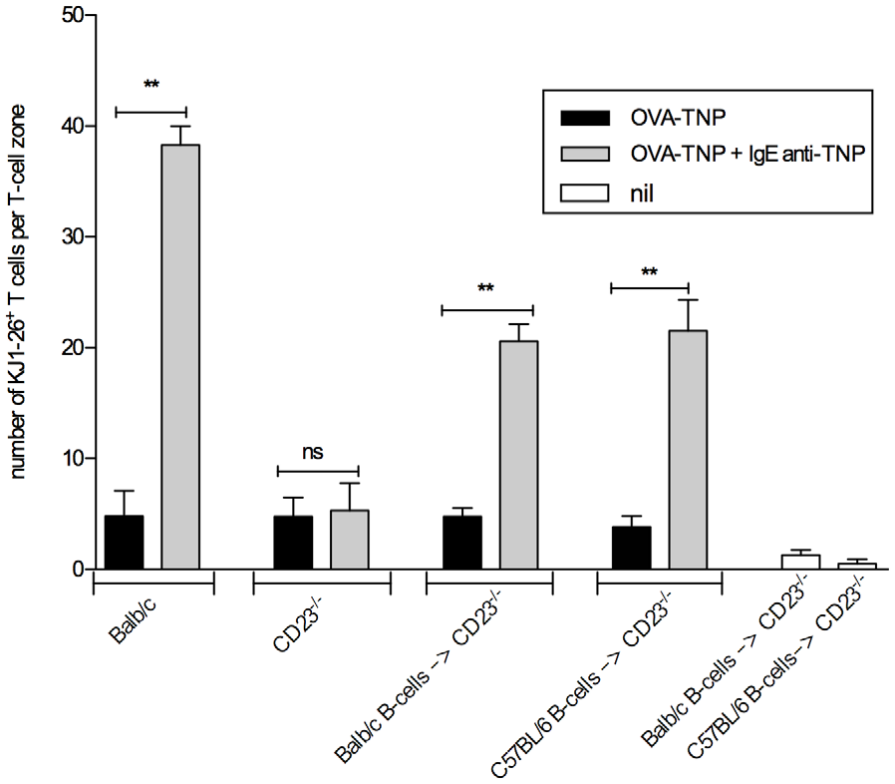
MHC-II incompatible B cells rescue IgE-mediated enhancement of T cell proliferation in CD23^-/-^ mice. Mice were transferred with 5×10^6^ CD4^+^ DO11.10 T cells. The next day, indicated mice were transfused with B cells (spleen cells depleted of CD43^+^ cells on a MACS column; 98% CD19^+^) and subsequently immunized with 20 µg OVA-TNP, with or without 50 µg IgE anti-TNP. Three days later spleens were removed and the number of KJ1-26^+^ T cells in non-consecutive sections of T cell zones were analyzed by confocal microscopy (2–4 mice/group; 4–16 T cell zones/mouse; mean values ± SEM). Representative of two identical and one similar experiment.

IgE cannot enhance CD4^+^ T cell or antibody responses in CD23^-/-^ mice, but transfer of syngeneic wildtype B cells can rescue these functions [16]. In addition, bone marrow derived cells (B cells), but not stromal cells (FDC), from wildtype mice rescued the ability of IgE to enhance antibody responses in CD23^-/-^ mice [11]. Both observations demonstrate the requirement for CD23^+^ B cells in this system. T cell responses are MHC restricted. Therefore, should the effector function of CD23^+^ B cells be to present IgE-antigen to DO11.10 T cells, only MHC-II compatible B cells would rescue CD23^-/-^ mice. However, should the effector function merely be to transport IgE-antigen to the spleen, both MHC compatible and incompatible wildtype B cells would rescue the response. To investigate this, CD23^-/-^ mice on a BALB/c background (MHC-II^d^) were reconstituted with B cells from either C57BL/6 (MHC-II^b^) or BALB/c (MHC-II^d^). Coming from wildtype mice, these B cells were CD23^+^. All animals had been transferred with DO11.10 T cells on the previous day and were immunized with OVA-TNP alone or in complex with IgE on the day of the B cell transfer. OVA-specific T cell proliferation was assessed by flow cytometry of spleen cell suspensions (data not shown) or confocal microscopy of spleen sections (Figure 4). As expected, T cells in BALB/c mice (bar 1 and 2) responded well to IgE-antigen whereas T cells in CD23^-/-^ mice (bar 3 and 4) did not respond. Also as expected [16], T cell proliferation in CD23^-/-^ mice was rescued by transfer of MHC-II compatible B cells (bar 5 and 6). Interestingly, MHC-II incompatible B cells (bar 7 and 8) were equally efficient in rescuing T cell proliferation as were MHC-II compatible B cells. This observation strongly suggests that the role of CD23^+^ B cells in this system is to transport rather than to present IgE-antigen complexes. There was a possibility that B cells from MHC-II incompatible mice could induce alloreactive responses by the OVA-specific T cells. However, neither BALB/c nor C57BL/6 B cells, transferred to CD23^-/-^ mice which were left unimmunized, were able to induce proliferation of DO11.10 T cells (Figure 4, bar 9 and 10).

## Discussion

The aim of the present study was to find out whether IgE-mediated enhancement of CD4^+^ T cell responses *in vivo* is caused by increased uptake and presentation of IgE-antigen by CD23^+^ B cells or whether it is more likely that these cells merely transport IgE-antigen to B cell follicles, where other cells take over and present the antigen to T cells. In summary, we were unable to find evidence for presentation of IgE-antigen complexes by CD23^+^ B cells *in vivo*. This is based on several experimental approaches. Using *ex vivo* antigen presentation assays we found (i) that CD19^-^, but not CD11c^−^ spleen cells, taken from wildtype mice 4 h after immunization with IgE-antigen, could induce proliferation of specific CD4^+^ T cells (Figure 1A), (ii) that positively selected CD11c^+^ spleen cells could induce proliferation of specific CD4^+^ T cells (Figure 1B), and (iii) that spleen cells taken from diphtheria toxin-treated CD11c-DTR mice (where CD11c^+^ cells are conditionally depleted) 4 h after immunization with IgE-antigen, lost their ability to induce proliferation of specific CD4^+^ T cells (Figure 1C). In a second approach we demonstrated that IgE-antigen did not induce T cell proliferation *in vivo* in diphtheria toxin-treated CD11c-DTR mice (Figure 3). Finally, both MHC-II compatible and MHC-II incompatible CD23^+^ B cells could rescue the specific T cell response to IgE-antigen complexes in CD23^-/-^ mice (Figure 4). As mentioned in the introduction, we have previously shown that IgE-complexed antigen can be found on peripheral B cells 10 minutes after immunizaiton and in the splenic B cell follicles (on the surface of follicular B cells) 30 minutes after immunization [15]. This finding demonstrated a new way for antigen to enter the follicles, namely attached via IgE to CD23^+^ recirculating B cells. It also provided a new possible explanation to why CD23^+^ B cells are required for IgE-mediated enhancement of antibody and CD4 T cell responses. Supported by in vitro findings this effect had been believed to be caused by B-cell mediated antigen presentation to T cells following uptake of IgE-antigen via CD23 [21], [22], [23], [24], [25]. The current data are suggestive of a scenario where CD23^+^ B cells transport IgE-antigen to the spleen where the antigen is captured and presented to CD4^+^ T cells by CD11c^+^ cells. Although we do not directly demonstrate the delivery of antigen from B cells to CD11c^+^ cells, we find that this is the explanation that best fits available data. In the CD11c-DTR mice, a gene that encodes the human diphtheria toxin receptor fused to green fluorescent protein (GFP) is inserted under the control of the CD11c promoter [36]. The promoter was initially considered specific for dendritic cells, but closer investigation has revealed activity also in alveolar macrophages, splenic marginal zone and metallophilic macrophages, a subset of activated T cells, NK cells and plasmablasts (reviewed in [40]). Nevertheless, we find it likely that the predominant cells presenting IgE-antigen to CD4^+^ T cells are dendritic cells. We cannot exclude involvement of splenic macrophages but the other CD11c^+^ cells are not antigen presenting cells. Dendritic cells are efficient APC and are able to trigger naïve T cells, a capacity not always found amongst B cells [32], [33], [34]. This has indeed been one of the arguments against the idea that B-cell mediated antigen presentation explains IgE-mediated enhancement. Mouse dendritic cells have not been found to express CD23. The observation that pure B cells can rescue the response to IgE-antigen in CD23^-/-^ mice [16], (Figure 4) corroborates this and shows that although CD11c^+^ cells are required, they do not act via CD23.

We do not yet know how IgE-antigen is transferred from B cells to CD11c^+^ cells. Intercellular exchange of proteins can occur in several different ways, e.g. via exosomes or trogocytosis [41]. Since dendritic cells are present at the boundary between T and B cell zones and also in B cell follicles [42], there should be ample opportunity for delivery of the IgE-antigen complexes from B cells to dendritic cells. The simplest explanation is that the presence of IgE-antigen on the B cell surface triggers neighboring dendritic cells to take up antigen via pinocytosis or endocytosis. Alternatively, exosomes containing CD23 and IgE-antigen bound to it, secreted by B cells after endocytosis of surface bound CD23 [43], may be very palatable to nearby dendritic cells. Antibody-antigen-complement complexes are transported on marginal zone B cells, expressing high levels of complement receptor 1 and 2, from the marginal zone to B cell follicles where they are delivered to FDC [44], [45]. Apart from B cells, FDC are the only cells in B cell follicles that express CD23. However, FDC do not seem to be involved in IgE-mediated enhancement, shown by the fact that B cells alone rescue the response in CD23^-/-^ mice [16] (Figure 4) and that bone marrow chimeras with CD23^-^ FDC respond well to IgE-antigen [11]. Therefore, a similar role for FDC in capturing IgE-antigen from follicular B cells as they have in capturing antigen-antibody-complement from marginal zone B cells, is unlikely. Instead our data suggest an alternative route for antigen handling where IgE-antigen complexes are transported by peripheral CD23^+^ B cells to B cell follicles where they are delivered to dendritic cells. Although we are not aware of any previous examples of antigen delivery from B cells to dendritic cells, the reverse has been observed [46], [47].

Several laboratories, including our own, have demonstrated that B cells via CD23 can take up and present IgE-antigen to T cells *in vitro* [21], [22], [23], [24], [25]. Nevertheless, the data presented here strongly argue against that B-cell-mediated antigen presentation explains why IgE is such a potent enhancer of T cell responses *in vivo*. The *ex vivo* assay (Figure 1) differs from previous *in vitro* studies in that the APC are only able to interact with the antigen while they are still in the animal - no further antigen is added to the cultures. Allowing cells and IgE-antigen to incubate *in vitro* may facilitate for CD23^+^ B cells to take up the complexes, as compared to the *in vivo* situation where the effective antigen concentration is smaller and the B cells are mobile. In addition, antigen transportation is not required *in vitro* and the ability of B cells to endocytose IgE-antigen via CD23 therefore may become predominant. This is an additional example of discrepant results obtained from *in vivo* and *in vitro* studies of the antibody feedback regulation system. IgG-mediated suppression of erythrocyte responses was independent of FcγRIIB *in vivo* but not *in vitro* [48], [49] and complement-tagged antigen could be taken up via complement receptors 1 and 2 on B cells and presented to T cells *in vitro* [50], [51], [52] but not *in vivo* [53].

Apart from its role in IgE-mediated enhancement of immune responses, CD23 is a negative regulator of IgE production. This is evidenced by increased IgE levels in CD23 knockout mice [54] and in mouse strains where CD23 is mutated [55], [56] as well as by reduced IgE production in transgenic mice overexpressing CD23 [57]. Interestingly, IgE-mediated enhancement works well in mice overexpressing CD23, showing that the negative and positive regulatory effects of CD23 are not mutually exclusive [25]. This is also what would be expected if the negative effect is caused by changes in B cell signaling whereas the positive effect is caused by transportation only.

Another interesting function of CD23 is to mediate transepithelial transport of IgE-antigen across intestinal barriers and across respiratory epithelium [20], [58], [59]. This effect is dependent on the CD23b isoform, whereas IgE-mediated enhancement of *in vivo* antibody responses is dependent on CD23a [12]. Nevertheless, it is an interesting parallel that IgE-antigen complexes can be transported bound to CD23 both on enterocytes [58], [59], airway epithelial cells [20] and B cells [15].

CD23 is known as the low affinity receptor for IgE, but does in fact have a dual affinity ranging from 2−7×10^6^ M^−1^ to 2−7×10^7^ M^−1^ [60]. The higher affinity is associated with the oligomeric, membrane bound form of the receptor [60] and up to 80% of peripheral B cells have recently been shown to be preloaded with IgE bound to CD23 [61], [62]. Presumably this IgE is sampled from the total IgE in the individual, thus reflecting the antigen experience of the host, and would enable rapid capture and transport of many different antigens by CD23^+^ B cells. One argument against a significant role of IgE in immune regulation has been that the serum levels are exceedingly low. However, the observations that B cells can be preloaded with IgE [61], [62] suggest that they may have acquired high enough concentrations of relevant IgE to mediate e.g. antigen transport regardless of a low serum concentration at the time of antigen entry. CD23 is a C-type lectin, a family of receptors known to act as pattern recognition receptors recognizing carbohydrate structures on pathogens [63]. Therefore, CD23 most likely can bind directly to certain carbohydrates expressed on pathogens. This may be an additional, IgE-independent, way for CD23^+^ B cells to transport antigen.

In summary we have presented data supporting the idea that peripheral CD23^+^ B cells capture IgE-antigen and transport it to splenic B cell follicles where antigen is delivered to dendritic cells which subsequently present it to T cells. The data argue against a role for CD23^+^ B cells in presenting IgE-antigen to T cells *in vivo*.
